# Technical Details of Surgical Treatment of a Severely Displaced Sternal Fracture

**Published:** 2019-04

**Authors:** Farshid Salehi, Shanay Niusha, Seyed Reza Saghebi, Seyed Morteza Razavi, Mohammad Behgam Shadmehr

**Affiliations:** 1 Tracheal Diseases Research Center (TDRC), National Research institute of Tuberculosis and Lung Diseases (NRITLD), Shahid Beheshti University of Medical Sciences, Tehran, Iran,; 2 Atieh Hospital, Tehran, Iran.

**Keywords:** Sternum, Surgery, Chest wall, Fracture

## Abstract

Sternal fracture is an uncommon injury, which is managed conservatively in most patients. In case of failure of non-surgical management or severely displaced fractures, open reduction and internal fixation should be considered. In this case report, we present the technical details of open reduction and internal fixation for a severely displaced sternal fracture in a bicyclist.

The sternal fracture was successfully treated, and the patient benefited from the rapid control of symptoms, early mobilization, and good cosmetic outcome.

**Conclusion:**

Open surgical treatment of a sternal fracture, when indicated, can be performed safely, with rapid control of symptoms, low risk of non-union, and good cosmetic outcome.

## INTRODUCTION

The most common cause of sternal fracture is direct blunt trauma to the anterior chest wall in motor vehicle accidents (1, 2). Sternal fracture is an uncommon injury (1), which is usually associated with other chest injuries, such as myocardial contusion, cardiac rupture, cardiac tamponade, pulmonary contusion, hemopneumothorax, and spinal injuries (1, 2). Chest pain aggravated by breathing and coughing, and localized tenderness is the most common clinical manifestations (2).

The diagnosis of sternal fracture is mainly made by history taking, physical examination, plain lateral chest X-ray, or chest CT scan when indicated (1, 2). After ruling out or stabilizing the associated injuries, the majority of patients, including those with non-displaced or mildly displaced fractures, are managed conservatively by painkillers, mucolytics, and chest physiotherapy (2). Patients with moderate to severe displacement may require surgical interventions for the management of their symptoms or cosmetic problems (3).

## CASE SUMMARIES

**Figure 1.**
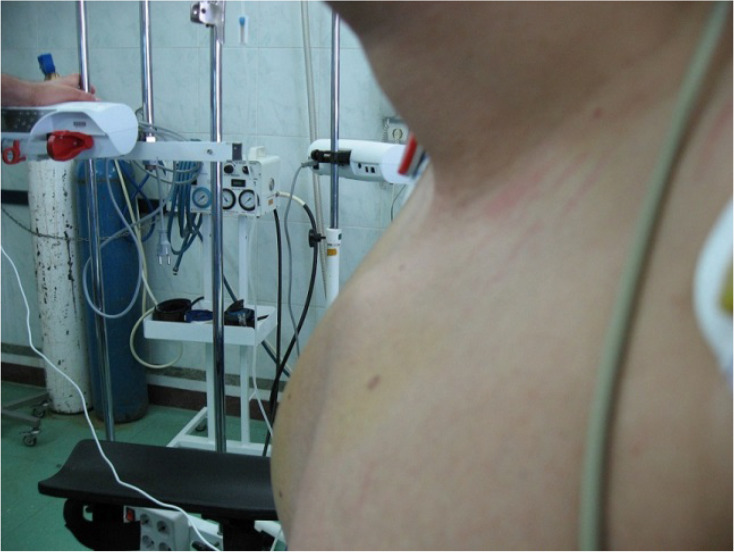
The patient with visible sternal deformity

**Figure 2.**
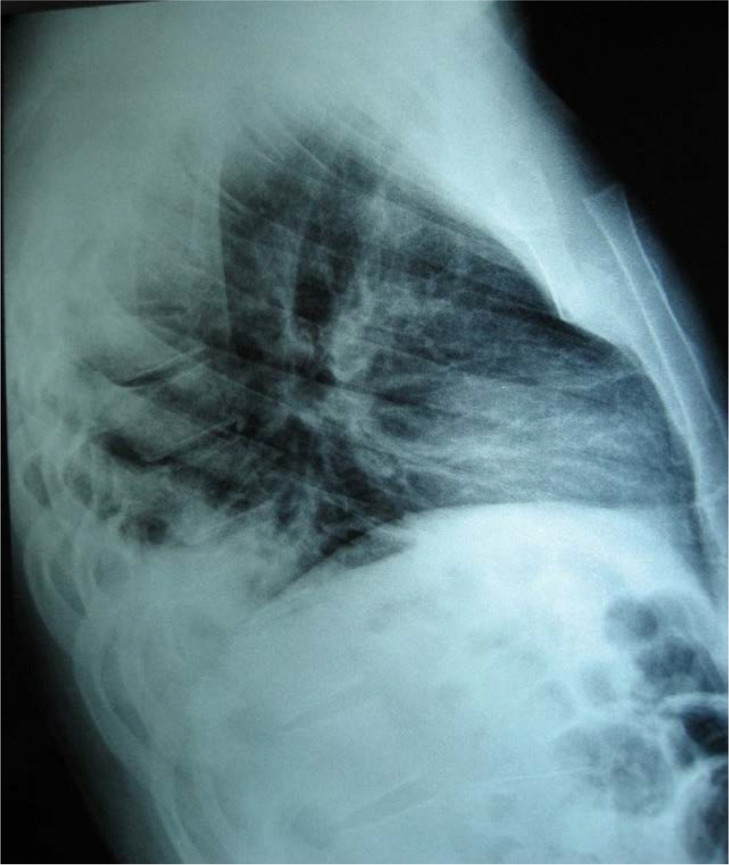
Patient’s lateral CXR

**Figure 3.**
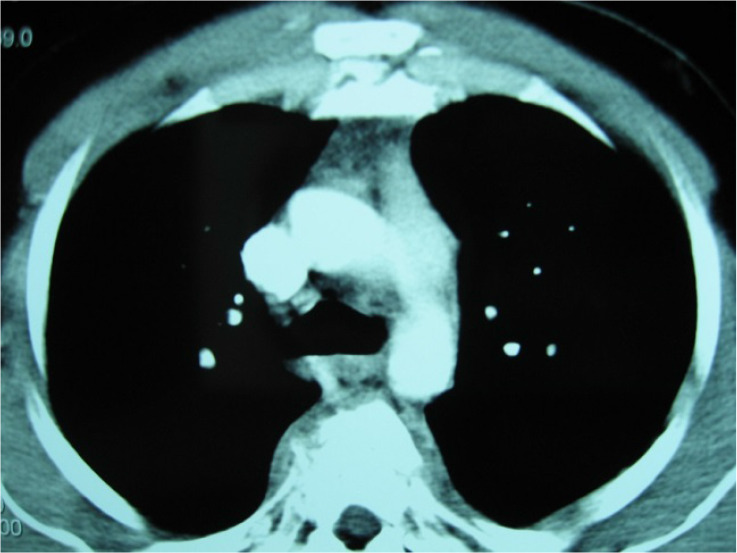
Patient’s chest CT scan

A 31-year-old male bicyclist fell off his bicycle and fractured his sternum severely. There were no associated injuries other than some facial bruises and a mild right-sided hemothorax, which was not treated in his first hospital admission. He had been discharged from the hospital and recommended to only use painkillers and wait for the spontaneous union of the fractured sternum. He later visited our hospital as an outpatient because of pain, dyspnea, and chest wall deformity (Figure 1). His lateral chest X-ray (Figure 2) and CT scan (Figure 3) showed a severely displaced and overriding fractured sternum.

The open surgical treatment was recommended to the patient and he consented to undergo surgery. The procedure was initiated with a midline incision over the upper two-thirds of the sternum. The pectoralis major muscles were dissected laterally to expose the sternum. The fracture line was identified right below the angle of Louis. There was a two-week-old callus, which was first resected. The space behind the sternum was then bluntly dissected with a finger through the suprasternal notch. Subsequently, the second rib cartilages, as well as a small part of the left third cartilage, were resected with preservation of both internal mammary vessels. A small wedge osteotomy was then performed at the distal part of the fracture line to facilitate reduction. The anesthesiologist was asked to fully paralyze the patient.

Reduction was performed by the surgeon and his assistant with forceful traction of tapes, which had been passed around the rib cartilages above and below the fracture line. The reduced sternum was then fixed with two parallel seven-hole plates. Three holes of each plate above the fracture line and three holes below the fracture line were pre-drilled and measured. Next, 12 sets of 3-5 mm cancellous screws were inserted, while the posterior plate of the sternum was guarded by the surgeon’s finger. The length of the screws ranged from 12 to 18 mm (7 bicortical and 5 unicortical screws). Intraoperative fluoroscopy confirmed the well-reduced sternum and the appropriate length of the screws. The pectoralis major muscle flaps were then advanced to the midline, and the wound was repaired in anatomic layers, without any drainage catheter.

**Figure 4.**
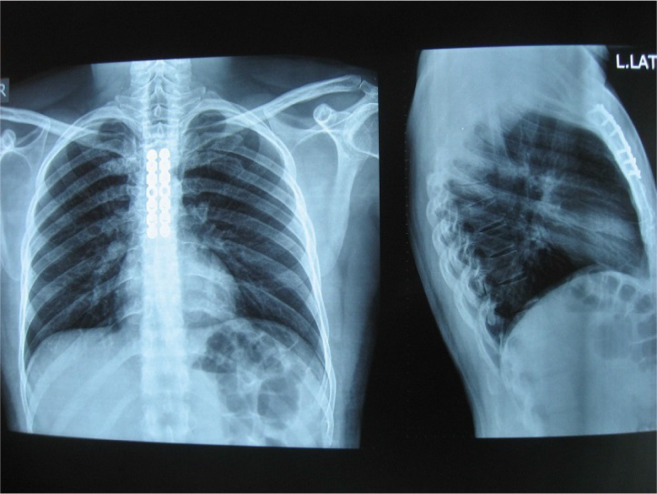
Follow-up CXRs after one year

The postoperative period was uneventful, except for a low-grade fever, which terminated after the insertion of a right-sided chest tube and drainage of 300 cc of the blood on the fourth postoperative day. The patient was discharged on the eighth postoperative day and returned after two weeks for a follow-up visit. He reported no further consumption of pain medications and expressed his satisfaction with the cosmetic outcome and early return to work. He returned to the hospital after one year and asked for plate and screw removal. The chest X-rays showed a well-healed sternum (Figure 4). Accordingly, the two plates and 12 screws were removed. Overall, the postoperative course was uneventful. He was also in good condition three years after the first surgery.

## DISCUSSION

Sternal fracture rarely occurs as an isolated injury and is usually associated with injury to other organs. Management of sternal fracture is not usually an emergency situation, except in occasional emergency surgeries for the associated injuries (2). There is a general consensus to primarily manage all non-displaced or mildly displaced sternal fractures conservatively (1–3). Although this approach is associated with the higher risk of atelectasis, pneumonia, malunion, non-union, and osteomyelitis, surgeons are now convinced to choose this non-surgical approach as the first step in the majority of cases, as it eliminates the need for surgery and has good clinical outcomes for many patients (1–3). On the other hand, open surgical reduction should be considered in patients with severely displaced fractures, as well as those with failure of conservative management due to malunion or non-union, and those with recurrence of chest pain, dyspnea, or chest deformity (3, 4).

There are different methods for fixing the fracture site after open surgical reduction. Although there are reports about steel wire suturing, the risk of malunion and subsequent complications is high (5). However, internal fixation by plates and screws not only allows for earlier mobilization but also leads to a more rapid and better control of patients’ symptoms (6, 7). There are different types of plates, which have been used and reported in the literature (3, 4, 7). We do not believe that the outcomes of surgery would be significantly different by using different types as long as the fracture is reduced perfectly and fixed tightly. Nonetheless, some technical issues should be kept in mind for better outcome.

If the callus is developed, it must be resected until reaching the fresh bleeding edge of the bone (7). Vascularization of the sternum should also be protected by minimal dissection and preservation of internal mammary vessels (4). Moreover, the reduction of fractured ends must be as accurate as possible, and any bone defect should be managed by bone grafts (8). For optimal reduction, it may be necessary to resect the costal cartilage, resect the wedge of the normal bone at one side, use tapes or bone clips for traction, and have the patient in a full-paralyzed anesthetic state (3). We preferred two parallel plates with three holes on each side to achieve better fixation and avoid interference with a possible median sternotomy in the future. Also, screws should be precisely selected for each hole, based on the thickness of the bone.

When there are no concerns about vascular injuries behind the sternum, bicortical screws are preferred due to their biomechanical advantage in providing better stabilization. However, we must always shield the posterior segment by a malleable retractor or surgeon’s finger. We preferred the latter in this study because the surgeon could feel the tip of the screw right after it egressed the posterior plate.

In conclusion, accurate open surgical treatment of a sternal fracture, if indicated, can lead to early mobilization, rapid control of symptoms, early return to work, lower risk of non-union, and good cosmetic outcome.
